# Efficient Interruption of Infection Chains by Targeted Removal of Central Holdings in an Animal Trade Network

**DOI:** 10.1371/journal.pone.0074292

**Published:** 2013-09-12

**Authors:** Kathrin Büttner, Joachim Krieter, Arne Traulsen, Imke Traulsen

**Affiliations:** 1 Evolutionary Theory Group, Max Planck Institute for Evolutionary Biology, Plön, Germany; 2 Institute of Animal Breeding and Husbandry, Christian-Albrechts-University, Kiel, Germany; University of Zaragoza, Spain

## Abstract

Centrality parameters in animal trade networks typically have right-skewed distributions, implying that these networks are highly resistant against the random removal of holdings, but vulnerable to the targeted removal of the most central holdings. In the present study, we analysed the structural changes of an animal trade network topology based on the targeted removal of holdings using specific centrality parameters in comparison to the random removal of holdings. Three different time periods were analysed: the three-year network, the yearly and the monthly networks. The aim of this study was to identify appropriate measures for the targeted removal, which lead to a rapid fragmentation of the network. Furthermore, the optimal combination of the removal of three holdings regardless of their centrality was identified. The results showed that centrality parameters based on ingoing trade contacts, e.g. in-degree, ingoing infection chain and ingoing closeness, were not suitable for a rapid fragmentation in all three time periods. More efficient was the removal based on parameters considering the outgoing trade contacts. In all networks, a maximum percentage of 7.0% (on average 5.2%) of the holdings had to be removed to reduce the size of the largest component by more than 75%. The smallest difference from the optimal combination for all three time periods was obtained by the removal based on out-degree with on average 1.4% removed holdings, followed by outgoing infection chain and outgoing closeness. The targeted removal using the betweenness centrality differed the most from the optimal combination in comparison to the other parameters which consider the outgoing trade contacts. Due to the pyramidal structure and the directed nature of the pork supply chain the most efficient interruption of the infection chain for all three time periods was obtained by using the targeted removal based on out-degree.

## Introduction

In the last decade, tremendous theoretical advances have been made in epidemiology on networks [1]–[4]. So far, such studies have implicitly focused mostly on the transmission of human diseases [5]–[8]. In more recent years, this kind of network analysis has also been increasingly applied to evaluate the risk of disease transmission through animal movements in the livestock industry. Most of these studies have focussed on analysing the structure of trade networks via animal movements and comparing trade networks of different time periods [9]–[15]. In order to utilise these insights, one has to infer how the spread of disease can be controlled by appropriately changing the network structure in the early phase of an epidemic. To understand the network resilience to the removal of parts of the network, percolation is an important concept [16]–[18]. The underlying idea is to remove a certain fraction of nodes until the network breaks apart [3], [19]–[22]. One practical example of a percolation process is the vaccination of animals. If an animal is vaccinated against a disease, it cannot transmit this disease to other animals. From an epidemiological perspective, this individual is removed from the network. This does not only prevent the animal from being infected, but it also can interrupt the chain of infection such that a further spread to other animals is prevented [3]. Nodes can be removed in different ways: at random or successively regarding their rank of different centrality parameters, e.g. in-degree and out-degree, ingoing and outgoing infection chain, betweenness centrality or ingoing and outgoing closeness centrality. Typically, it makes sense to remove highly central nodes first.

The aim of this study was to understand how a targeted removal of nodes can affect a pyramidal animal trade networks and be superior to a random removal of nodes based on the analysis of changes in the network structure of the movement data of a pork supply chain of a producer community in Northern Germany. We compared the random removal of holdings to the targeted removal of holdings according to their ranking of specific centrality parameters. By evaluation of the structural changes in the network topology, it was possible to identify the optimal method to decompose the network into fragments and thereby interrupt the chain of infection.

## Materials and Methods

### Data, Network Construction and Classification of the Time Periods Analysed

Data on pig movements from a producer community in Northern Germany were obtained from an observation period between 1^st^ of June 2006 to 31^st^ of May 2009. The data contain the date of the movement, the codes of the holdings of origin and destination, as well as the batch size and the type and age group of the delivered livestock. A total of 15,372 directed animal movements were recorded between 658 holdings; each of the animal movements had one specific supplier and one specific purchaser. Movements of pigs were registered at group level by animal batches.

**Figure 1:**
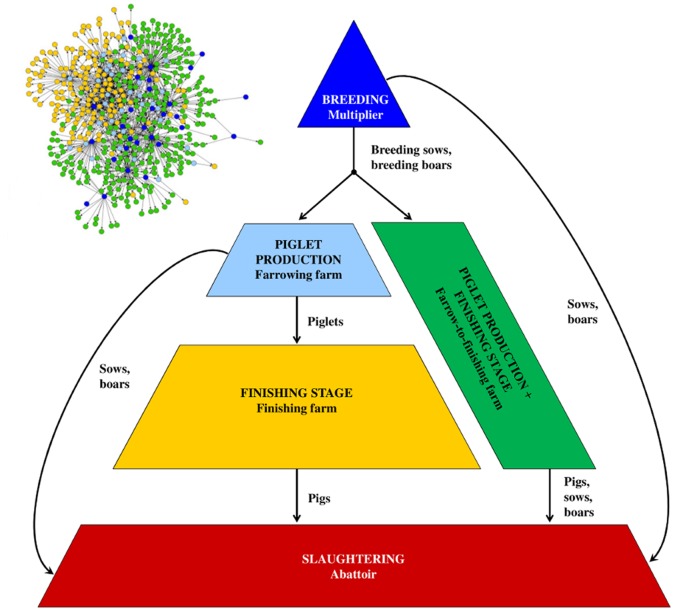
Pyramidal structure and holding type classification including the delivered livestock of the pork supply chain. The small network in the left corner of the figure illustrates the pork supply chain of the aggregated three-year network. Abattoirs are excluded from the network illustration.

The data were separated into different time periods: one accumulated three-year network, three yearly networks and 36 monthly networks. If there were multiple trade contacts between two holdings throughout the analysed time periods, they were aggregated into a single one. By means of number and age groups of the transported animals, the production type was classified into five different holding categories: multipliers, farrowing farms, finishing farms, farrow-to-finishing farms and abattoirs. As shown in Figure 1, this reflects the classical pork supply chain, which has a pyramidal structure with the multipliers at the beginning. These multipliers produce breeding sows and breeding boars which are then used in the farrowing farms for the production of piglets. Further, the piglets produced are brought to the finishing farms where they are fattened until they reach their final weight. Then they are transported to the abattoir. The second path in this pyramidal structure shows the farrow-to-finishing farms. In this holding type, piglets are produced and raised until they have gained their final weight. In this way, the two functions of farrowing farms and finishing farms are combined in one holding type. Besides the connections illustrated in Figure 1, cross-connections can also exist.

Abattoirs and their related movements were excluded from the network due to the dead-end characteristic of this holding type for the transport of live pigs and an inclusion of them would lead to an overestimation of the potential risk for other holdings close to the end of production chain for spreading an infection. Therefore, the network size of the three-year network changed to 483 holdings and 4,635 animal movements, which were aggregated to 926 group movements [15]. The yearly networks had on average 322 (319 to 323) holdings and 1,545 (1,522 to 1,571) animal movements, aggregated to 449 (431 to 468) group movements. The monthly network had on average 129 (107 to 148) holdings and 427 (359 to 479) animal movements aggregated to 114 (93 to 134) group movements [14].

### Analysis of Network Parameters

The analysis was performed separately for each time period. A short definition of the centrality parameters used is provided in Table 1. Beside the well-described degree, betweenness and closeness centrality, we measured also the ingoing and outgoing infection chain. These parameters can be seen as extensions to the degree centrality. It measures the direct as well as the indirect trade contacts considering the chronological order of the animal movements. Due to the directed nature of the pork supply chain we have to distinguish between the ingoing and the outgoing infection chain, meaning the trade contacts which lead to a certain holding and the trade contacts which had left a certain holding [9], [23], [24]. The general network properties and the above-mentioned centrality parameters are reported in [15] for the three-year network and in [14] for the yearly and the monthly networks. In this study, the robustness of these networks was analysed in order to infer an optimal strategy to interrupt and terminate the outbreak of a disease.

**Table 1:** Description of parameters used in network analysis for the characterisation of animal movements.

Parameter	Definition	References
In-degree	Number of trade partners which deliver animals to a specific holding	[3]
Out-degree	Number of trade partners which receive animals from a specific holding	[3]
Ingoing infection chain	Number of direct and indirect trade contacts which lead to a specific holding taking the chronologicalorder of the contacts into account	[9]
Outgoing infection chain	Number of direct and indirect trade contacts which originate at a specific holding taking thechronological order of the contacts into account	[23], [24]
Betweenness	The betweenness centrality measures the extent to which a holding lies on paths betweenother holdings	[47]
Ingoing closeness	Mean distance from all other reachable holdings to one specific holding	[47]
Outgoing closeness	Mean distance from one holding to all other reachable holdings	[47]

### Percolation Theory and Network Resilience

One process which can be applied to connect the network structure with its functions is the so-called percolation process. Percolation theory, used in statistical physics to describe phenomena such as fluids moving through porous media or conductivity in random networks [16]–[18], can be used to study the decomposition of a network by removing nodes according to different selection criteria. The nodes can be removed with uniform probability or successively based on the rank of their centrality parameters, i.e. the targeted removal of the most central holdings. The changes in the network structure by means of percolation processes can be represented based on the size of the largest weakly connected component, depending on the fraction of removed holdings. Weakly connected components were chosen in this case due to the acyclic nature of the networks under investigation [14], [15]. Two holdings are part of the same weakly connected component if at least one path through the network connects these two holdings. The paths are allowed to go either way along any link [25].

Percolation theory can be used to connect the network topology with the processes taking place in a specific network to explore network resilience. Many real-world networks, e.g. the Internet or collaboration networks, are very tolerant when nodes are removed at random [3]. This means that the general topology and the global connectedness of the network remain even if a lot of nodes are removed. However, the same networks show a high vulnerability regarding the targeted removal of highly central nodes. This property is typical for right-skewed distributions of centrality parameters, i.e. the majority of nodes has a very low value for the centrality parameters, but there are few nodes with a very high centrality [3]. The targeted removal of these highly central holdings results in a rapid change in the network structure and can lead to a fast fragmentation of the network. Identifying the most efficient method of fragmenting the network structure can thus help to optimize the intervention and control strategies during an epidemic. Splitting the network into small pieces interrupts the chain of infection and a further spread of disease can be prevented efficiently. The change in the size of the largest network component is not only an indicator of network resilience, but it also gives the opportunity to assess the maximum possible epidemic size in the network under investigation [26].

To compare possible measures to stop epidemics in an animal trade network, we compared the random removal of holdings to a successive removal based on their ranking of specific centrality parameters. This allowed us to identify the most suitable centrality parameter for such a procedure. Furthermore, the optimal combination with the highest reduction in the largest network component for a small number of holdings was explored. This allowed an assessment of the differences between the targeted removal and the optimal combination of removed holdings in the area of fewer removed holdings.

#### Random removal of holdings

As a base case, we considered the successive random removal of holdings. As this procedure can lead to very different outcomes, we performed 1,000 independent removal procedures and assessed the average size of the largest remaining component and its distribution. Alternatively, holdings were removed at random, but differentiated by their holding types. Again, every removal procedure was averaged over 1,000 independent realisations.

#### Targeted removal of holdings based on the ranking of centrality parameters

In this case, the holdings were successively removed from the network based on the rank of the calculated centrality parameters (in- and out-degree, ingoing and outgoing infection chain, betweenness centrality and ingoing and outgoing closeness centrality). If there was more than one holding with the same value for a centrality parameter, all such holdings were removed in a single step.

#### Optimal combination of removed holdings

In this case, we removed all possible combinations of holdings from the network with one, two or three holdings from the network and calculated the size of the largest network component for each combination. The combination with the highest reduction in the size of the largest network component was considered as the optimal combination of removed holdings.

### Statistical Analysis

Statistical analysis was performed using SAS® statistical software package [27]. All computations concerning the network properties and the percolation process were performed using the Python module NetworkX [28].

For each network, the holdings were removed at random with 1,000 independent realisations. For each removal step, we calculated the median values as far as the 10^th^, 25^th^, 75^th^ and 90^th^ percentile for the size of the largest network component. In the plots, we illustrated the median as dotted line, the values between the 25^th^ percentile and the 75^th^ percentile as a dark grey area and the values between the 10^th^ and the 90^th^ percentile as a light grey area. A linear regression was performed to quantify the effect of the removal procedure with the procedure PROC REG of the SAS® statistical software package [27]. We then fitted the slope of the median random removal for a fraction of removed holdings of less than 50%.

## Results

### Random Removal of Holdings

**Figure 2:**
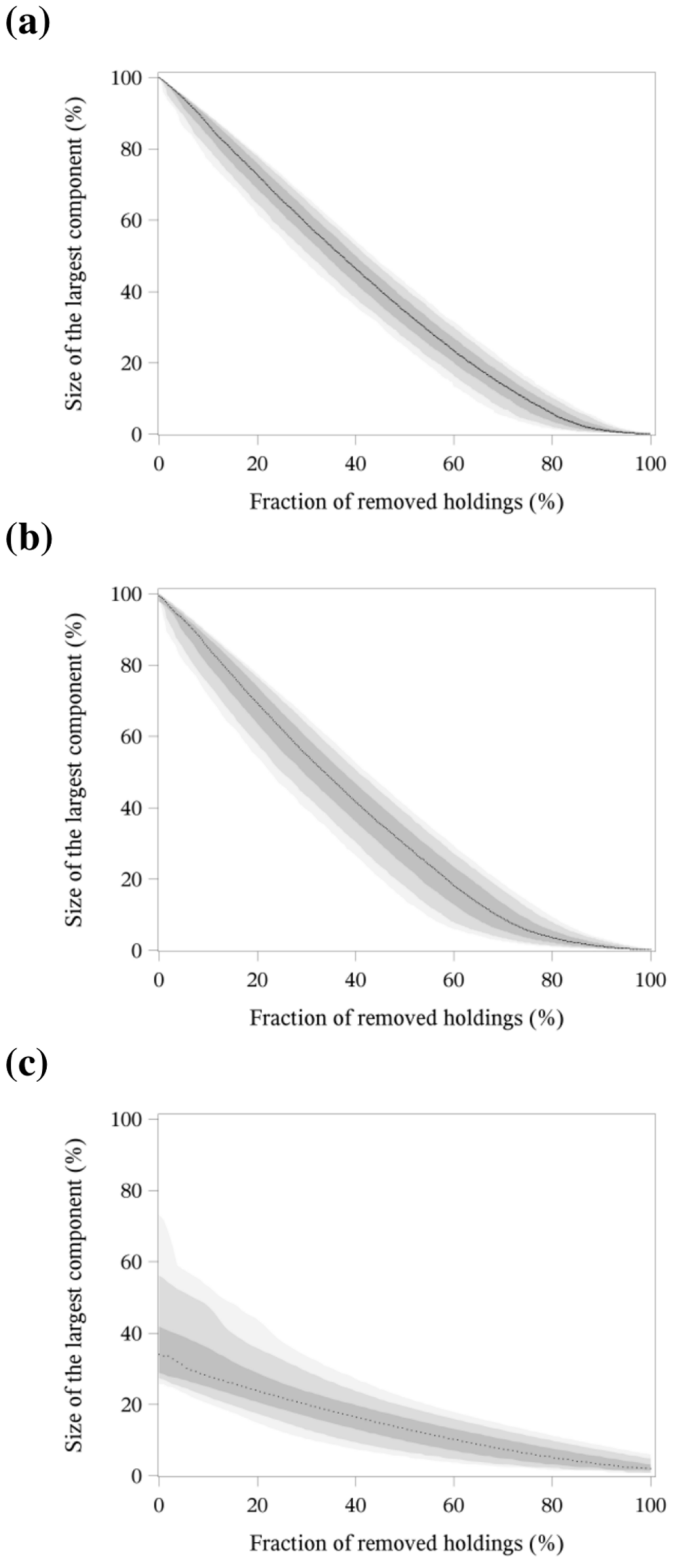
Random removal of holdings in the three-year network (a), the yearly networks (b) and the monthly networks (c). Size of the largest network component depending on the fraction of removed holdings.

Figure 2 illustrates the random removal of holdings in the three-year network, the yearly and the monthly networks. In order to compare the procedure between the three time periods, a linear regression was performed to fit a slope of the median values calculated over all iterations for less than 50% of removed holdings (Table 2). Here, also a differentiation by holding type was carried out. All fittings showed significant results (p<0.05). The random removal of holdings for the three-year network (Figure 2a) and the yearly networks (Figure 2b) had similar curve shapes with a slope of −1.34 and −1.42. A totally different curve shape was obtained for the monthly networks (Figure 2c). Here, the average slope of the linear regression function was substantially lower (a = −0.40). As expected, the range of the values for the slope of the linear regression was much wider for monthly networks than for the yearly networks.

**Table 2:** Slope of the linear regression (a) of the iterations of the random removal for less than 50% of removed holdings [range].

	a	R^2^	p
Total three-year network	−1.34	0.99	<0.0001
Multipliers	−10.63	0.99	<0.0001
Farrowing farms	−3.13	0.99	<0.0001
Finishing farms	−1.00	1.00	<0.0001
Farrow-to-finishing farms	−1.07	0.99	<0.0001
Yearly networks	−1.42[−1.49 to −1.40]	0.99	<0.0081
Multipliers	−12.20[−14.4 to −11.82]	0.99	<0.0001
Farrowing farms	−2.38[−2.63 to −2.42]	0.99	<0.0001
Finishing farms	−1.01[−1.03 to −1.01]	0.99	<0.0001
Farrow-to-finishing farms	−1.09[−1.11 to −1.08]	0.99	<0.0001
Monthly networks	−0.40[−1.35 to −0.30]	0.99	<0.0083
Multipliers	−2.95[−14.4 to −1.00]	0.96	<0.0001
Farrowing farms	−0.71[−4.04 to −0.20]	0.98	<0.0001
Finishing farms	−0.30[−2.29 to −0.07]	0.98	<0.0001
Farrow-to-finishing farms	−0.40[−1.45 to −0.32]	0.99	<0.0001

Multipliers showed the lowest values for the slope of the linear regression for all three observed time periods and therefore the most rapid reduction in the size of the largest network component. The random removal of multipliers for the three-year network and the yearly networks had similar values for the slope with −10.63 and −12.20, whereas the monthly networks showed only an average slope of −2.95 for the multipliers, but a wider range. In the three-year network and the yearly networks the multipliers were followed by the farrowing farms with a slope of −3.13 and −2.38. All other holding types had only a small slope of the linear regression and therefore no or only a small reduction in the size of the largest network component.

### Targeted Removal of Holdings based on the Rank of Centrality Parameters

**Figure 3:**
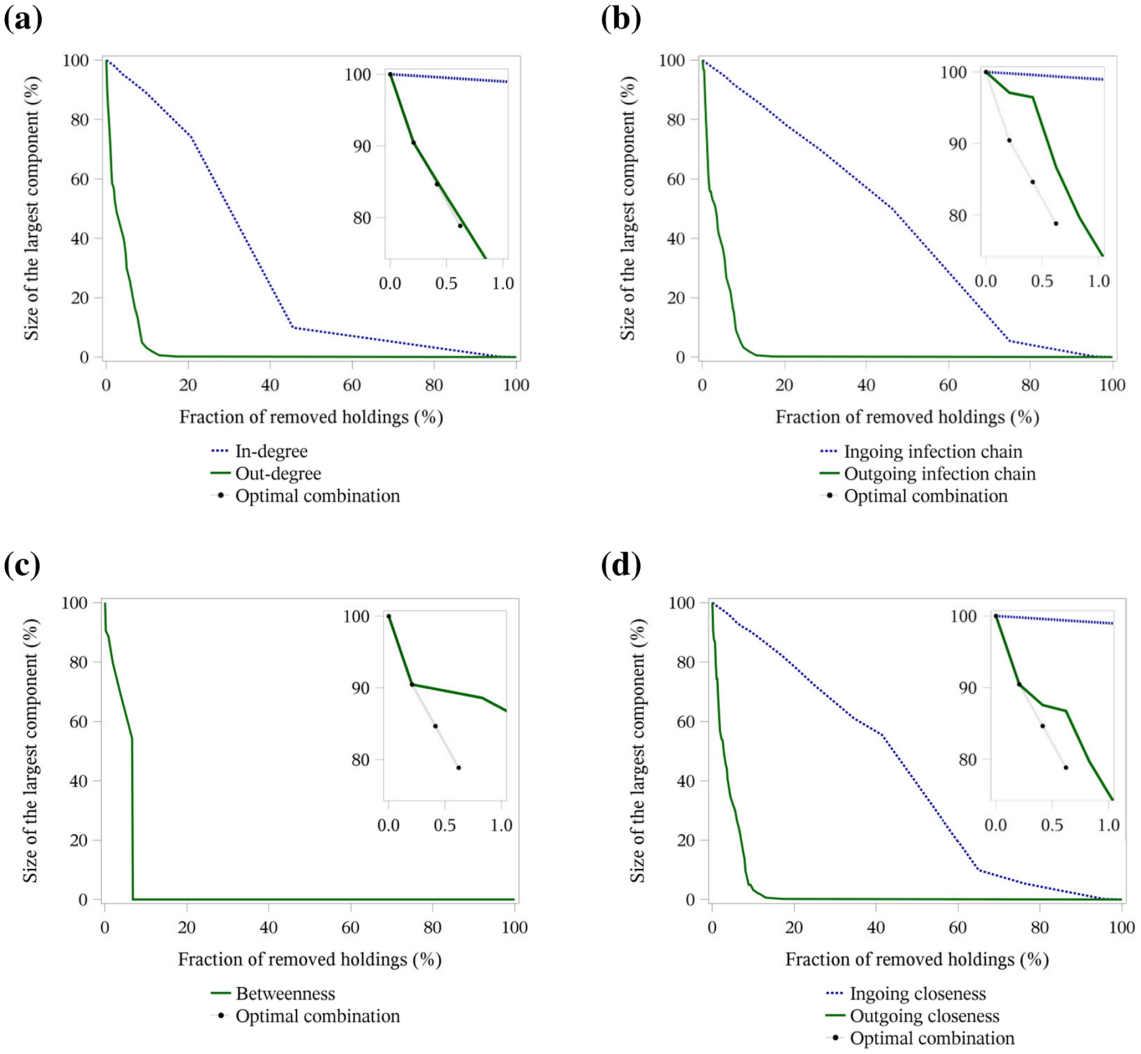
Targeted removal of holdings in the three-year network. Size of the largest network component depending on the fraction of removed holdings regarding the centrality parameters in- and out-degree (a), ingoing and outgoing infection chain (b), betweenness centrality (c) and ingoing and outgoing closeness centrality (d). The number and proportion of removed holdings to achieve a reduction in the size of the largest component by more than 75% for the total three-year network is shown in Table 3. The inset of each figure shows the optimal combination of the first three removed holdings in comparison to the targeted removal of holding based on centrality parameters.

**Figure 4:**
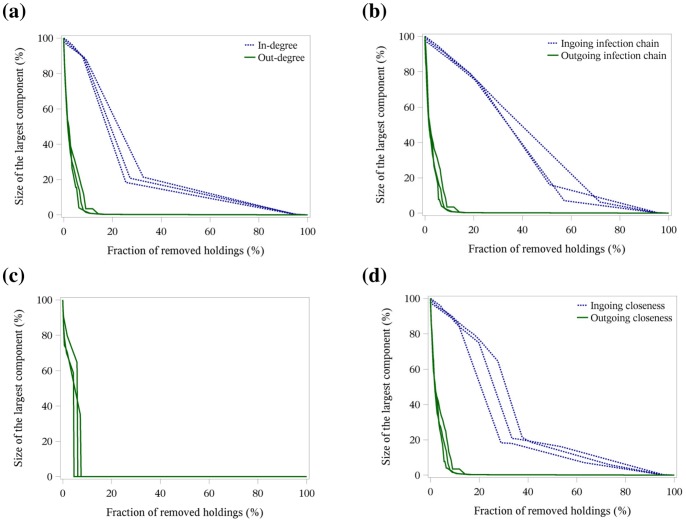
Targeted removal of holdings in the yearly networks. Size of the largest network component depending on the fraction of removed holdings regarding the centrality parameters in- and out-degree (a), ingoing and outgoing infection chain (b), betweenness centrality (c) and ingoing and outgoing closeness centrality (d). The number and proportion of removed holdings to achieve a reduction in the size of the largest component by more than 75% for the yearly networks is shown in Table 4.

**Figure 5:**
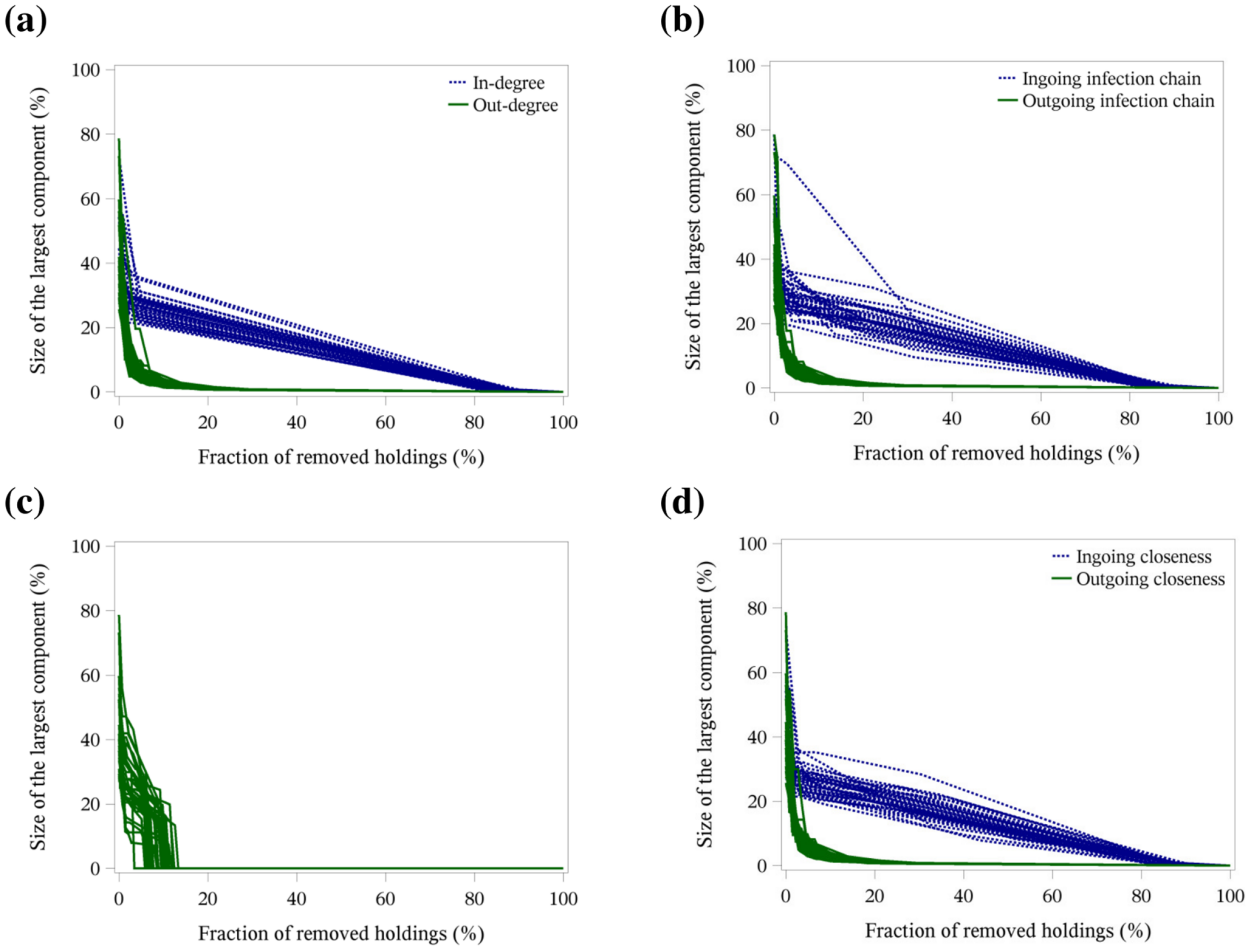
Targeted removal of holdings in the monthly networks. Size of the largest network component depending on the fraction of removed holdings regarding the centrality parameters in- and out-degree (a), ingoing and outgoing infection chain (b), betweenness centrality (c) and ingoing and outgoing closeness centrality (d). The number and proportion of removed holdings to achieve a reduction in the size of the largest component by more than 75% for the monthly networks is shown in Table 5.

Figure 3, 4 and 5 illustrate the targeted removal of holdings with regard to the rank of the centrality parameters in-degree and out-degree (a), ingoing and outgoing infection chain (b), betweenness centrality (c) and ingoing and outgoing closeness centrality (d) for the three-year network (Figure 3), the yearly networks (Figure 4) and the monthly networks (Figure 5). The figures show that the removal of holdings based on parameters describing outgoing trade contacts (out-degree, outgoing infection chain and outgoing closeness centrality) induced a rapid change in the network structure, i.e. less than 20% of the holdings had to be removed to reduce the size of the largest network component close to zero. Also, the successive removal of holdings with the highest betweenness centrality resulted in a fast reduction in the size of the largest network component. These observations held for all time periods under investigation.

In contrast, for the three-year network, the removal of holdings based on the centrality parameters in-degree showed for the first 20% of removed holdings nearly no change in the size of the largest network component (Figure 3a). If the removal was based on ingoing infection chain and ingoing closeness centrality, more than 60% of the holdings had to be removed to achieve a substantial reduction in the size of the largest network component (Figures 3b and 3d). For the yearly networks, more than 40% of the holdings had to be removed based on in-degree and ingoing closeness centrality to reduce the size of the largest network component by more than 80% (Figures 4a and 4b). For the ingoing infection chain, more than 60% of the holdings had to be removed to achieve the same reduction (Figure 4d). In the monthly networks, only a few holdings (less than 10%) had to be removed to obtain a size of the largest component of 20% of the network size. In contrast to the centrality parameters based on the outgoing trade contacts or the betweenness centrality, the decrease stagnated and nearly all holdings had to be removed to destroy the largest network component (Figures 5a, 5b and 5d).

To evaluate the influence of the different holding types on the reduction in the size of the largest network component, Table 3, 4 and 5 show the number and the proportion of removed holdings differentiated by their holding type in the three-year network, the yearly and the monthly networks to reduce the size of the largest network component by more than 75% by targeted removal.

**Table 3:** Three-year network.

Parameter	Number (proportion in %) of removed holdings to reduce the largest network component by more than 75%				
	Total	Multiplier	Farrowing farm	Finishing farm	Farrow-to-finishing farm
In-degree	220 (46)	5 (17)	24 (71)	77 (50)	114 (43)
Out-degree	31 (6)	16 (55)	11 (32)	–	4 (1)
Ingoing infection chain	362 (75)	5 (17)	29 (85)	129 (84)	199 (75)
Outgoing infection chain	32 (7)	18 (62)	9 (26)	–	5 (2)
Betweenness	32 (7)	8 (28)	16 (47)	–	8 (3)
Ingoing closeness	314 (65)	5 (17)	29 (85)	113 (74)	167 (63)
Outgoing closeness	32 (7)	17 (59)	9 (26)	–	6 (2)

Number and proportion of removed holdings to reduce the size of the largest network component by more than 75% by targeted removal depending on the ranking of specific centrality parameters.

For holding types, the proportion refers to the number of this specific holding type in the three-year network.

**Table 4:** Yearly networks.

Parameter	Number (proportion in %) [range in %] of removed holdings to reduce the largest network component by more than 75%				
	Total	Multiplier	Farrowing farm	Finishing farm	Farrow-to-finishing farm
In-degree	91 (28)	1 (7)	11 (36)	36 (37)	43 (24)
	[25]–[33]	[6]–[10]	[35]–[36]	[33]–[46]	[22]–[27]
Out-degree	17 (5)	9 (51)	5 (16)	–	3 (2)
	[4]–[6]	[50–53]	[7]–[21]		[1]–[3]
Ingoing infection chain	193 (60)	2 (10)	20 (65)	71 (74)	101 (57)
	[51–72]	[6]–[13]	[45–81]	[65–79]	[40–73]
Outgoing infection chain	18 (5)	11 (61)	4 (12)	–	3 (2)
	[5]–[6]	[56–67]	[7]–[16]		[1]–[3]
Betweenness	19 (6)	4 (25)	10 (31)	–	5 (3)
	[4]–[7]	[20]–[33]	[25]–[39]		[2]–[4]
Ingoing closeness	107 (33)	1 (7)	11 (37)	49 (52)	45 (25)
	[29]–[38]	[6]–[10]	[35]–[39]	[45–60]	[22]–[29]
Outgoing closeness	17 (5)	10 (57)	4 (12)	–	3 (2)
	[4]–[6]	[55–60]	[7]–[16]		[1]–[3]

Number and proportion of removed holdings to reduce the size of the largest network component by more than 75% by targeted removal depending on the ranking of specific centrality parameters. For holding types, the proportion refers to the number of this specific holding type in the yearly networks.

**Table 5:** Monthly networks.

Parameter	Number (proportion in %) [range in %] of removed holdings to reduce the largest network component by more than 75%				
	Total	Multiplier	Farrowing farm	Finishing farm	Farrow-to-finishing farm
In-degree	109 (85)	3 (30)	10 (52)	37 (99)	59 (95)
	[81–90]	[11]–[44]	[33–79]	[96–100]	[89–99]
Out-degree	5 (4)	4 (48)	0 (1)	–	0 (0)
	[1]–[8]	[22–89]	[0–20]		[0–2]
Ingoing infection chain	108 (84)	3 (29)	10 (51)	37 (97)	59 (93)
	[34–90]	[0–44]	[32–79]	[39–100]	[37–99]
Outgoing infection chain	5 (4)	4 (50)	0 (1)	–	0 (0)
	[2]–[8]	[25–86]	[0–20]		[0–2]
Betweenness	10 (8)	2 (25)	5 (28)	–	3 (4)
	[3]–[13]	[11]–[44]	[11]–[47]		[0–11]
Ingoing closeness	107 (83)	3 (29)	10 (51)	36 (96)	58 (93)
	[44–90]	[0–44]	[29–79]	[41–100]	[56–99]
Outgoing closeness	5 (4)	4 (49)	0 (1)	–	0 (0)
	[1]–[8]	[22–86]	[0–20]		[0–2]

Number and proportion of removed holdings to reduce the size of the largest network component by more than 75% by targeted removal depending on the ranking of specific centrality parameters. For holding types, the proportion refers to the number of this specific holding type in the monthly networks.

The most rapid reduction in the size of the largest network component in the three-year network (Table 3 and Figure 3) was obtained by the successive removal of holdings regarding their out-degree, outgoing infection chain, betweenness centrality and outgoing closeness centrality. In total, only 7% of the holdings had to be removed from the network to reduce the size of the largest network component by more than 75%. The holding type which had to be removed the most for the centrality parameters out-degree, outgoing infection chain and outgoing closeness centrality was the multipliers with 59%, followed by the farrowing farms with 28%. The farrowing farms had to be removed the most only for the betweenness centrality; in this case 47%, followed by the multipliers with 28%. No finishing farms had to be removed to achieve a reduction by more than 75%.

The successive removal of holdings based on the parameters which consider the ingoing trade contacts was not efficient. In total, about 62% of the holdings had to be removed for the parameters in-degree, ingoing infection chain and ingoing closeness centrality to reduce the size of the largest network component by more than 75%. The holding type which had to be removed the most for the above-mentioned centrality parameters was the farrowing farms with 80%, followed by the finishing farms with 69% and finally the farrow-to-finishing farms with 60%. The holding type which had to be removed least was the multipliers with 17%.

For the yearly networks, the most rapid reduction in the size of the largest network component, i.e. only about 5% of the holdings had to be removed to achieve a 75% reduction of the largest network component, could be obtained by successive removal based on the parameters out-degree, outgoing infection chain, betweenness centrality and outgoing closeness centrality (Table 4 and Figure 4); this was a similar result compared to the three-year network. The distribution of the different holding types also showed similar results. For the parameters out-degree, outgoing infection chain and outgoing closeness centrality, multipliers had the highest percentage with about 56%, followed by farrowing farms with 13%. As seen in the results of the three-year networks, the order of the most removed holding types changed for the betweenness centrality with 31% of farrowing farms followed by 25% of multipliers. Also, in the yearly networks, no finishing farms had to be removed for the above-mentioned centrality parameters.

Considering the parameters which measured the ingoing trade contacts, 40% of the holdings on average had to be removed to reduce the size of the largest network component by more than 75%. The proportions of removed holding types were nearly in the same range and order compared to the three-year network, only finishing farms with 54% and farrowing farms with 46% changed their ranks, followed by the farrow-to-finishing farms with 35% and finally the multipliers with 8%.

For the monthly networks, similar results were obtained as for the three-year network and the yearly networks (Table 5 and Figure 5). The successive removal of holdings depending on the centrality parameters out-degree, outgoing infection chain, betweenness centrality and outgoing closeness centrality induced the most rapid reduction in the size of the largest network component. Only 5% of the holdings had to be removed to achieve a reduction of the size of the largest network component by more than 75%. But the percentage for the betweenness centrality with 8% was nearly twice as high as for the above-mentioned centrality parameters. Another difference between the results of the three-year network and the yearly networks is that almost exclusively multipliers had to be removed for the parameters out-degree, outgoing infection chain and outgoing closeness centrality with a percentage of 49%. For the betweenness centrality, the proportion of removed holding types was divided almost equally between farrowing farms with 28% and multipliers with 24%.

By successive removal of holdings regarding the parameters in-degree, ingoing infection chain and ingoing closeness centrality in the monthly networks, about 84% of the holdings had to be removed to achieve a reduction in the largest component by more than 75%. The holding types which had to be removed the most for the above-mentioned centrality parameters were the finishing farms with 97% and the farrow-to-finishing farms with 94%, followed by the farrowing farms with 51%. The holding type which had to be removed least was the multipliers with about 29%.

### Optimal Combination of Removed Holdings

Table 6 and the insets in Figure 3 show the improvement in network decomposition by the removal of the optimal combination of the first three holdings in comparison to the targeted removal of holdings based on the ranking of specific centrality parameters for all three time periods. The results of the targeted removal by the centrality parameters based on the ingoing trade contacts differentiated the most from the optimal combination of the first three removed holdings. In the three-year network, the difference was 20.6%, in the yearly networks 30.7% and in the monthly networks 19.2%. The smallest difference between the three-year network and the optimal combination was obtained for the removal by out-degree with 1.0%, followed by outgoing infection chain and outgoing closeness centrality with 7.9% and betweenness centrality with 10.4%. In the yearly networks, the removals by out-degree and outgoing closeness centrality led to sizes of the largest cluster of about 2% above the optimal strategy, followed by outgoing infection chain with 5.6% and betweenness centrality with 11.2%. For the monthly networks the smallest difference to the optimal combination was the removal by out-degree, outgoing infection chain and outgoing closeness centrality with 1.5%. Removal by betweenness centrality converged to the removal by the centrality parameters based on the ingoing trade contacts. Furthermore, the range of the values increased markedly from the yearly networks to the monthly networks.

**Table 6:** Improvement in % [range in %] of network decomposition (reduction in the size of the largest network component) by removal of the optimal combination of the first three holdings in comparison to the targeted removal of holdings regarding the calculated centrality parameters for the total three-year network, the yearly and the monthly networks.

Parameter	Three-year network	Yearly network	Monthly network
In-degree	20.5	30.7 [28.1 to 32.3]	19.2 [9.5 to 39.2]
Out-degree	1.0	1.7 [0.3 to 2.5]	1.4 [–2.5 to 18.7]
Ingoing infection chain	20.5	30.6 [27.9 to 32.3]	19.9 [7.9 to 59]
Outgoing infection chain	7.9	5.6 [0.3 to 12.9]	1.5 [–1.6 to 7]
Betweenness	10.4	11.2 [7.7 to 17.3]	15.7 [0 to 35.4]
Ingoing closeness	20.5	30.6 [27.9 to 32.3]	17.9 [9.5 to 32.1]
Outgoing closeness	7.9	2.0 [0 to 3.7]	1.7 [0 to 17.8]

If we look at the specific holdings, a single multiplier appeared in every optimal combination over all observed time periods.

## Discussion

Previous analyses of this network showed that it had a significant right-skewed distribution of the calculated centrality parameters for all observed time periods, which indicates a large heterogeneity [14], [15]. Other trade networks of animal movements have revealed similar patterns [9], [10], [13], [29]–[31], despite the fact the trade network strongly depends on the transported species. Such a distribution, with a majority of holdings having a very small centrality value and only few holdings with a very high centrality, has important implications for processes taking place in this kind of network, such as the spread of an epidemic. Due to the few highly central holdings, the network structure is very robust regarding the random removal of holdings. The probability of hitting the few highly central holdings is very low in this procedure; therefore, a lot of holdings have to be removed to destroy the network structure. But if these highly central holdings are removed in a targeted fashion, a rapid fragmentation of the network can be obtained [30], [32]–[34]. The prototypic example of such a phenomenon is the Internet, which has been shown to be very robust towards random removal of nodes, but highly vulnerable to targeted attacks [19]–[21].

### Random Removal of Holdings

The results of the random removal of holdings indicated that it is not an appropriate method to rapidly interrupt the chain of infection and stop the spread of an epidemic. This result is in agreement with similar work on other networks with a right-skewed distribution. This kind of network cannot be destroyed by the random removal of holdings [3]. For the linear regression, the fraction of less than 50% of removed holdings was chosen, since it is not desirable to remove a large part of the network in any practical application. During an epidemic, it is desirable to interrupt the chain of infection by only removing a small amount of holdings from the trade network – this is not achievable by the random removal of holdings in the present network.

### Target Removal of Holdings based on the Ranking of Centrality Parameters

The targeted removal of holdings based on out-degree, outgoing infection chain, betweenness centrality and outgoing closeness centrality was an efficient way to decompose the trade network into fragments. This property was obtained from all analysed time periods. Other studies have shown similar results for undirected networks. According to [35], the targeted removal of highly connected holdings, so-called hubs, is a very effective method for disease control. Due to the pyramidal structure and the directed nature of the pork supply chain, the most rapid fragmentation of the network could be obtained by the targeted removal of holdings based on out-degree. The out-degree measures only the direct outgoing trade contacts of a holding. Hence, also holdings located in the middle of the pork supply chain can have a high value for this parameter. Due to the fact that not only holdings at the beginning of the pork supply chain are removed (cf. outgoing infection chain and outgoing closeness centrality), the removal of holdings based on out-degree had a higher potential to decompose the network. Nevertheless, multipliers and farrowing farms were the most central holding types in our network. Their removal led to a rapid fragmentation of the network, meaning that only a small amount of holdings had to be removed from the network. During an epidemic, monitoring these specific holding types is of particular importance.

The betweenness-based removal of holdings showed also a fast reduction in the size of the largest component. In other studies, similar results could be obtained [36], [37]. In the present network, especially the farrowing farm holding type had the highest betweenness centrality. In contrast to previous studies, the fast decomposition of the trade network based on the removal regarding the rank of the betweenness centrality did not imply that the trade network consists of different communities [36]–[40]. However, it can rather be explained by the pyramidal structure of the pork supply chain. Deleting the holdings at the second level of the production chain, i.e. farrowing farms, the trade network is interrupted. Between multipliers and finishing the farrowing farms build the link over which the most connections had to run.

The centrality parameters in-degree, ingoing infection chain and ingoing closeness centrality were less suitable for a rapid fragmentation of the network structure. Although their distributions were right-skewed, the values obtained showed a smaller range than the other centrality parameters. One underlying reason for this is the pyramidal structure of the pork supply chain under investigation. The vast majority of animal movements went through the system in a directed way. Therefore, the holding types at the end of the pork supply chain had the highest values for these centrality parameters. But removing the holdings at the margin of the network cannot affect disease spreading within the network. Thus, the size of the largest network component is only reduced by a few holdings and not separated into parts. Instead, this happens for example by removing holdings with a high betweenness centrality. This also explains the fact that the removal by in-degree showed a faster reduction in the size of the largest network component than the removal by ingoing infection chain and ingoing closeness centrality. The centrality parameters ingoing infection chain and ingoing closeness centrality take the whole chain of contacts into account, also the indirect contacts. Therefore, the holdings at the end of the pork supply chain, i.e. finishing farms and farrow-to-finishing farms, had the highest values of these two centrality parameters. But, as mentioned above, removing these holdings at the end of the pork supply chain had only little impact on the network structure due to the pyramidal structure and directed nature of the present trade network.

The results of [14] illustrated that in contrast to [26] most variations of the centrality parameters considering the outgoing trade contacts and the betweenness centrality could be observed for the holdings with the highest values. But this does not mean that the holdings with the highest ranks changed during time, only their values changed. This implies that from an epidemiological perspective the control measures have to focus on out-degree, outgoing infection chain, betweenness centrality or outgoing closeness centrality. In the present trade network these parameters remained relatively stable over all observed time periods, meaning that even the three-year network could be used to implement control strategies regarding the calculated centrality parameters. The differences between the results of [26] and the present study can be explained by the pyramidal structure of the pork supply chain in contrast to the cattle trade network. In the pork supply chain, a minority of the holdings is located at the beginning of the production chain, i.e. multipliers and farrowing farms. And this minority had the highest values for the parameters considering the outgoing trade contacts. In other studies, similar results were obtained for pig movement data [9], [10], [13], [41]. Thus, it is important to distinguish which kind of species is transported since clear differences occurred in the structures analysed. According to [10], the pig trade network is a specific network and should be mainly compared with other pig trade networks. Only for the centrality parameters considering the ingoing trade contacts similar results as in [26] could be found. However, due to the fact that the removal based on the centrality parameters considering the ingoing trade contacts were less effective, these parameters were not worth considering for the implementation of control strategies.

### Optimal Combination of Removed Holdings

Table 6 and Figure 3 show the results of the optimal combination of the first three removed holdings. For practical purposes, an optimal strategy is typically out of reach, but we can assess which method for the removal of nodes is closest to the optimal removal. The smallest difference between the above-mentioned removals and the optimal combination was obtained by targeted removal based on outgoing trade contacts. Although the targeted removal of holdings by betweenness centrality also resulted in a rapid fragmentation of the trade networks, the difference was larger in this case. One reason for this is the pyramidal structure of the pork supply chain under investigation. Therefore, there were no holdings which built bridges between two parts of the network. This effect became more evident the shorter the observed time period was. In the monthly networks, the removal based on the betweenness centrality showed nearly the same difference as the removal based on in-degree, ingoing infection chain and ingoing closeness centrality.

If we look at the specific holdings which were part of the optimal combination, one multiplier appeared in every optimal combination of all observed time periods. As the results of [14] showed, especially the ranking of the holdings remained stable over time considering the parameters based on the outgoing trade contacts or the betweenness centrality. That is the reason why the optimal combination of removed holdings consisted of these similar compositions. Table 6 shows that the optimal combination of the first three removed holdings can be worse than the targeted removal by out-degree and outgoing infection chain. This results from the procedure in which the holdings are removed from the network. As mentioned in the materials and methods section, if there were holdings with the same values for specific centrality parameters, all of them were removed in a single step. In contrast, the removal of the optimal combination was done one holding after the other.

### Summary

The removal of holdings regarding the rank of the centrality parameters out-degree, outgoing infection chain, betweenness centrality and outgoing closeness centrality induced a rapid fragmentation of the networks under investigation. This network characteristic can be used to interfere quickly in the first phase of an epidemic, e.g. by targeted culling of high-risk holdings or selective vaccination of holdings in a surveillance zone, to control and to contain the outbreak. In case of an epidemic it is important to act fast. With the help of network analysis and percolation theory it is possible to identify holdings which are more likely to spread or to contract an infection and to know the behaviour of the trade network by the targeted removal of holdings. This cannot only help to improve the control and surveillance strategies during an epidemic, it can also help to put up strategies to prevent the introduction into the supply chain, e.g. via movement restrictions for specific holding types or selected vaccination of susceptible animals [30].

The present study focused on the removal of holdings from the trade network, i.e. by selected vaccination or culling, the so-called site percolation. In future studies, it would be of great interest to study the removal of the contacts between different holdings, the so-called bond percolation [3]. This would correspond to the trade restrictions put up as a control strategy in the case of a disease outbreak. In this way, not only the holding types can be classified into high or low risk holdings, but also the contacts between the holdings.

Besides this further exploration of the properties of our network, our work also highlights the need for further theoretical studies: Most network-based models for epidemic spreading are implicitly developed for human diseases, leading to undirected networks [42]–[46]. However, trade networks in animal production are typically characterised by directed links. Also in the case of dynamic networks, the establishment of new links in such directed trade networks is more cumbersome than in the case of undirected networks [1].

## Conclusion

The centrality parameters of our trade network had a right-skewed distribution, which has important consequences for network resilience. The random removal of holdings in all three observed time periods did not lead to a rapid fragmentation of the network structure, even though the largest slope was obtained for multipliers. In contrast, by selective removal of the most central holdings, e.g. via selective vaccination or culling, the network structure decomposes and further disease spread can be prevented. Therefore, targeting highly central holdings is much more effective than the random removal of holdings. The targeted removal of holdings based on out-degree, outgoing infection chain, betweenness centrality and outgoing closeness centrality was a highly efficient method to interrupt the chain of infection during an epidemic. However, the removal by out-degree showed the most rapid fragmentation and did not differ substantially from the optimal removal of nodes. The reason for this is the pyramidal structure and the directed nature of the pork supply chain with the majority of the animal movements taking a directed path through the system. In contrast, the removal of holdings based on the rank of the centrality parameters in-degree, ingoing infection chain and ingoing closeness centrality is not an appropriate method to decompose the network structure. Knowledge of the structure of trade networks and their reaction to the removal of holdings or contacts can be used to optimise control strategies during an epidemic or to improve prevention measurements. We anticipate that control strategies which do not take the network structure into account are not as effective as the targeted removal of nodes, but more efficient than a random removal of nodes.
